# Long-term assessment of the self-purification potential of a technologically managed ecosystem: the Middle Iskar cascade

**DOI:** 10.1080/13102818.2014.923623

**Published:** 2014-08-26

**Authors:** Stilyana Lincheva, Yovana Todorova, Yana Topalova

**Affiliations:** ^a^ Department of General and Applied Hydrobiology, Faculty of Biology, Sofia St. Kliment Ohridski University, Sofia, Bulgaria

**Keywords:** hydrochemical and microbiological parameters, reservoirs, water quality

## Abstract

The Middle Iskar cascade is situated along the middle course of the Iskar River (Bulgaria) after the capital city Sofia and has three small hydroelectric power plants that were put into operation by the end of 2012. The aim of this study was to evaluate the self-purification potential of water in the reservoirs of these plants as an important and necessary condition for their ecological functioning. The assessment was made by hydrochemical parameters (dissolved suspended solids, insoluble suspended solids, total suspended solids, nitrites, nitrates, ammonium, phosphates, chemical oxygen demand, dissolved oxygen) and microbiological parameters (aerobic heterotrophic bacteria and bacteria growing in an Endo medium) and covered a period of three years (2010, 2011 and 2012). Standard methods were applied, mainly colorimetric and microbiological cultivation methods. The obtained results showed high levels of some of the tested indicators during 2012. In the section of the Middle Iskar cascade a high self-purification potential was observed in the reservoirs which maintain good water quality.

## Introduction

The world consumption of energy from renewable sources continued to increase in 2011 and 2012 despite the international economic crisis, the political instability and the decreasing support of some key markets.[1] Renewable energy reached up to about 19% from the world consumption of energy during 2011, the last year about which there is available information. Of these 19% approximately 9.3% are from the energy from traditional biomass. The usable heat energy from modern renewable sources engages 4.1%; the energy from water power plants is a little above 3.7% and 1.9% is for the wind, solar and geothermal energy.

Along with this, the latest evaluations show that at least 40% of the surface waters in the European Union are at risk of not achieving the aim of the Water Framework Directive (WFD) for 2015, which requires good ecological and chemical condition in terms of low levels of chemical pollution and healthy ecosystem.[2,3] Achieving good ecological condition of the Iskar River (the longest river in Bulgaria and the third in length of the water basin) is of key importance for achieving the aim of the WFD for the waters.[4] The most significant pollution of the Iskar River is in its middle course after passing the capital city Sofia. The pollution of Iskar, beginning several decades ago and continuing, though at slower rate until today, poses danger of risky situations worsening the quality of the waters threatening the ecosystem's health.[5]

The steady increase of the part of the renewable energy sources in the world is the reason of the construction of the Middle Iskar cascade situated along the middle stream of the Iskar River after Sofia. The cascade had three out of its nine small hydroelectric power stations (SHPPs) put into operation by the end of 2012. The first hydroelectric power plant put into operation in May 2008 is SHPP Lakatnik, a year after that SHPP Svrazhen began to function and in June 2012 the third power plant, Tserovo. Their effective functioning, however, meets some ecological problems. This part of the river has been accumulating pollutants of different origin for decades. In addition, most of the settlements in the area of the cascade do not have treatment facilities and their wastewater further deteriorates the water quality of the river. The small reservoirs of hydroelectric power plants are places where the river water slows its speed, leading to concentration and accumulation of different types of pollutants (e.g. organic and xenobiotic pollutants). Long-term assessment of the self-purification potential of the Iskar River in the area of the Middle Iskar cascade as an inseparable part of the river waters is essential for the environmental assessment of the river region.

The microbiological and hydrochemical parameters of the waters of the reservoirs in each of the three hydro power plants give information about the speed and the scale of the self-purification potential. The present study covered three consecutive years (2010, 2012, 2013) and was carried out in two seasons: high-flow and low-flow seasons. The aim of the study was to evaluate the self-purification potential of the waters in the reservoirs of the small hydro power plants as an important and necessary condition for their ecology and appropriate functioning.

## Materials and methods

**Figure 1.**
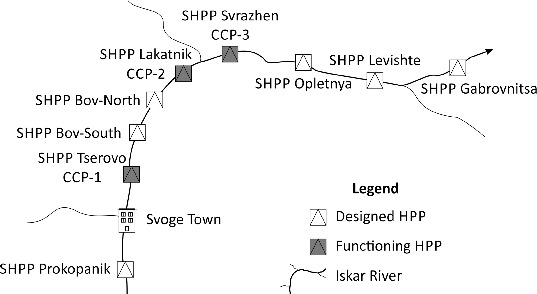
Location of the cascade of small hydropower plants and sampling stations.

The sites for taking samples are situated in the reservoirs of the three functioning hydroelectric power plants (Figure 1): Tserovo (near the wall of the reservoir), Lakatnik and Svrazhen (from the central part of the reservoirs). Two sample-takings per year were made in three consecutive years (2010, 2011 and 2012): in June, i.e. during a period of high water level, and in September, i.e. during a period of low water level. Water samples were taken with a Limnos water sampler from different depths: 0 m, 3.5 m, 7 m at Reservoir Tserovo; 0 m, 1 m, 2.5 m, 3.5 m at Reservoir Lakatnik; and 0 m, 2.5 m, 5 m at Reservoir Svrazhen.

Data shown in the figures represent mean values from those measured at the above-mentioned depths. This experimental design was based on our previous study which showed that the fluctuations of the hydrochemical and microbiological parameters on horizons are insignificant.[6] This gives us reason to claim that the micro reservoirs are polymictic and that is why below we present averaged data from different horizons. In the laboratory triplicate measurements (for all parameters discussed in the paper) were made for each sample from each depth.

The samples for microbiological studies were collected in sterile containers and stored in cool bags at 4 °С. All samples were transported to the laboratory within 4–6 h and immediately processed. The microorganisms were identified by means of routine cultivation methods used in hydro-microbiological practice.[7] Aerobic heterotrophic bacteria (AH) and the quantity of the bacteria growing on an Endo medium were studied.

After transportation, the water samples were filtered and the concentration of NH_4_^+^, NO_2_^−^, NO_3_^−^ and PO_4_^3−^ was measured colourimetrically.[8] The chemical and microbiological methods for testing the water are listed in Table 1.

**Table 1.** Chemical and microbiological methods used for testing the water in this study.

No.	Indicator	Method
(1)	Chemical oxygen demand (COD)	BNS 17.1.4.02-77
(2)	Dissolved oxygen, oxygen saturation	BNS EN 25814
(3)	Dissolved suspended solids (DSS)	BNS 17.1.4.04-80
(4)	Insoluble suspended solids (ISS)	BNS 17.1.4.04-80
(5)	NH_4_^+^	BNS ISO 7150-1
(6)	NO_2_^−^	BNS EN26777
(7)	NO_3_^−^	BNS ISO 7890-3
(8)	PO_4_^3−^	BNS EN1189
(9)	Aerobic heterotrophic bacteria	BNS ЕN ISO 6222:2002
(10)	Coliform bacteria	BNS 17336-93

Note: BNS: Bulgarian National Standard; EN: European Standards; ISO: International Organization for Standardization.

## Results and discussion

Rivers are key natural and ecological sites from a water management perspective. Plans are made for managing the rivers which are inseparable part of the strict requirements for the implementation and functioning of WFD.[4] The integrated management of the water resources in a regional and global aspect requires simultaneous and effective application of a number of multi-disciplinary approaches, one of the most important ones being the long-term monitoring of the quantity, quality and the ecological status of the natural and the anthropogenically influenced waters. The three-year study of the waters of the SHPPs as an inseparable part of the waters of the Iskar River is part of the long-term monitoring that has been going for seven years now in this section of the river. The dynamics of dissolved suspended solids (DSS), insoluble suspended solids (ISS), total suspended solids (TSS), of some hydrochemical parameters important for the transformation of carbon, nitrogen and phosphorous, as well as two key groups of microorganisms were studied.

The dynamics and variation of studied hydrochemical parameters depend on a combination of factors. Some of the main factors are the hydrological regime of the river system (the high-flow and low-flow seasons related to different quantity of water in the river), the status of the sediments and the incoming organic matter in the river (in different critical situations, e.g. torrential rains and floods, the sludge can rise and go in the waters, changing the concentration of the ionic forms there). An additional influence is exerted by the waste waters from the town of Svoge and the surrounding populated places that do not have wastewater treatment plants and burden the river with different types of pollutants. The biggest part of these belongs to pollutants of protein origin. That is why the concentrations of the ions strongly depend on the rate and scale of the biodegradation of protein-containing organic matter. It is degraded by means of basic processes such as hydrolysis, the first and the second phase of nitrification, each of which depends on a number of factors.

Last but not least, the concentration of the studied ions depends on the technological management of the Middle Iskar cascade. In the SHPP reservoirs, sediments are accumulated, containing organic and xenobiotic pollutants carried by the river. Upon the release of these sludges along the course of the river the concentration of the nutrients can change significantly. A unique ecological situation was registered during the high-flow season in 2011 when due to poor technological management of SHPP Lakatnik several meters of sediments were accumulated in the reservoir. The long stay of the sludge in the reservoir caused intensive anaerobic biodegradation processes, leading to release of different gases from the surface of the reservoirs. This in turn is related to influence on the processes of degrading organic matter in the waters, influence on the microbial communities and on the environment.[9]

### Dynamics of suspended solids

**Figure 2.**
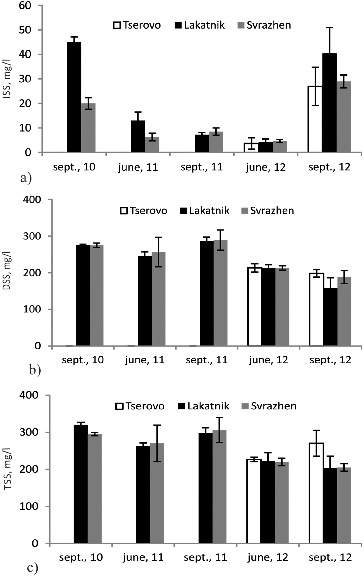
Dynamics of insoluble suspended solids (a), dissolved suspended solids (b) and total suspended solids (c) in the water of the reservoirs during the studied seasons.

The dynamics of ISS, DSS and TSS in the water of the reservoirs during the studied seasons are shown in Figure 2. ISS is a direct indicator for the quantity of insoluble matter in the waters of the reservoirs (the matter that has not settled down on the bottom or has been re-suspended from there). Our results showed that the concentration of ISS fluctuated with an overall trend towards its decrease from 2010 to 2012 (Figure 2(a)). The exception was the low-flow season in 2012 when in allthe three reservoirs the amount of ISS increased again. The possible reason could be the building activities on the SHPP Prokopanik located upstream, which caused an increase of the insoluble substances in the waters. The highest concentration of ISS was measured in the Lakatnik reservoir for almost all studied seasons and years. This is due to the fact that until SHPP Tserovo was put into exploitation (in June 2012) the Lakatnik reservoir was the first functioning SHPP along the river course. The slow-down of the water speed in the reservoir of the first SHPP most probably led to the measurement of the highest concentration of ISS. An alternative interpretation of the ISS results could be made based on the poor management of the sediments of the SHPPs registered in 2011. Figure 2(a) shows that the concentration of ISS in the reservoirs was low in 2011 in comparison to other years. This could probably be due to the long retention of the sediments in the reservoirs (without release), hence the lack of mass transfer and aeration which causes a large part of ISS to settle down on the bottom of the reservoirs.

On the other hand, the dynamics of the DSS (Figure 2(b)) show that in the same year, 2011, their concentration was the highest. This is probably due to a slow-down in the process of aerobic degradation, replaced by anaerobic processes in the sediments, the more difficult absorption of the dissolved substances from the microbial consortium due to change of the conditions in the water environment, which results in higher DSS concentrations in the waters. The lowest DSS values were observed in 2012, which again confirms the improvement of the water quality from 2010 to 2012. This improvement could have partly been due to the recommendations provided by the team for more frequent release of the sediments in the reservoirs and preventing risky situations such as the one described above.

The TSS include the organic particles and minerals that are transported in the water. TSS in the water can also be an indicator for land erosion that took place. The level of TSS can range from less than 5 mg/L to 30,000 mg/L or more and varies from one river to another.[10,11]

The dynamics of TSS (Figure 2(c)) and DSS in our study show that a big part of the organic matter in the waters was in a dissolved state and could be easily and quickly included in the self-purification biodegradation processes with correct technological management of the cascade of small hydro power plants. During periods of low water level, there was a greater discrepancy between the TSS and DSS values, which suggests that in calmer waters part of the dissolved organic matter settles down or becomes attached on the surfaces of the suspended substances.

### Forms of nitrogen

**Figure 3.**
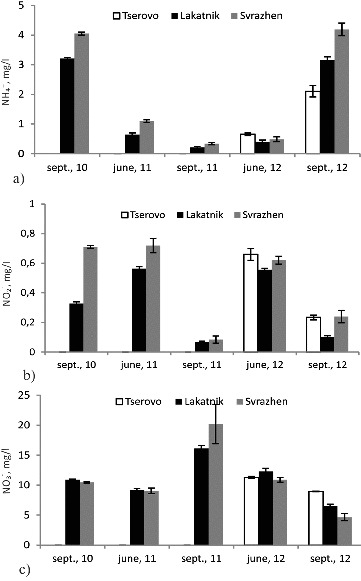
Dynamics of ammonium (a), nitrite (b) and nitrate ions (c) in the water of the reservoirs during the studied seasons.

The forms of nitrogen analysed in this study included ammonia, nitrite and nitrate (Figure 3). The dynamics of the ammonium ions concentration in the three reservoirs showed seasonal and annual variations (Figure 3(a)). The concentrations of ammonium ions are dependent on their sources such as livestock waste and fertilizers. Septic systems and improper disposal of household cleaning products containing ammonia are also sources of ammonia in the rivers and their reservoirs.[12] With the exception of the high-flow season in 2012, in the Svrazhen reservoir there were highest concentrations of ammonium ions. The low-flow seasons of 2010 and 2012 were characterized with considerably higher levels of these ions than the ones in the other studied seasons. It is possible that the lesser amount of waters in September may have caused the increase in the ion concentration. Figure 3(a) and 3(c) shows that from June 2011 to June 2012 including, nitrification processes prevailed in the waters of the reservoirs (low concentrations of ammonium ions and high ones of nitrates). In September 2010 and 2012, the nitrates decreased whereas the concentration of the ammonium ions increased with every sequential reservoir along the course, i.e. more intensive processes of ammonification were under way. Baurès et al. [13] observed that the carbon to nitrogen (C/N) ratio expressed from total organic carbon (TOC) and nitrate concentrations showed high values for extreme flows and particularly for very low-flow rates, generally in summer. Our study also showed that the concentration of nitrates (Figure 3(c)) was the highest during the low-flow season in 2011 in the Svrazhen reservoir. Nitrate ions had the lowest concentration in the same reservoir during the low-flow season in 2012, where their concentration decreased along the course. The reason for higher levels of nitrates in June 2012 than in September 2012 could be related to the fact that the seasonal pattern of NO_3_^−^ concentrations in running waters has shown a change in the last few years, with a tendency towards slightly lower leaching of NO_3_^−^ during the growing season.[14]

Similar to the ammonium ions, the nitrites (Figure 3(b)) had the highest concentration in the Svrazhen reservoir during all studied seasons except for the high-flow season in 2012 when their concentration was greater in the Tserovo reservoir. The lowest nitrite concentrations were measured during the low-flow season of 2011 when the lowest concentrations of ammonium ions and the highest concentrations of nitrates were observed. Considering the risky situation in 2011 again, we found the following: in June 2011 comparatively low concentrations of ammonium ions, high levels of nitrites and comparatively low ones of nitrates. This supports the idea that certain processes may have been inhibited as a result of poor management of the sediments in the reservoir. In this case the inhibition is at the second phase of the nitrification process, i.e. when the nitrites are transformed into nitrates. This could have caused the accumulation of nitrites and the increase in their concentrations. As early as the next sample-taking in September 2011 there were registered the lowest levels of ammonium ions and the highest concentration of nitrates. This shows that in September 2011 the nitrification processes in the reservoirs from the Middle Iskar cascade were more intense and the nitrogen in the water was presented mostly in the form of nitrates, i.e. the mineralization processes of N-containing organic matter were being brought to the end. This is probably due to release of the sediments after our summer sample-taking, the increase of aeration and the intensification of the biodegradation processes. The described situation about the forms of nitrogen in June 2011 was also observed in June 2012, whereas in September the same year the prevailing processes were not denitrification, but ammonification ones.

The results about the nitrogen forms show that the processes of degradation of N-containing organic matter are delicate and strongly dependent on the above-mentioned combination of factors: content of the incoming waters from the dwelling places around the river; technological management of the cascade of small hydro power plants; and the rate of biodegradation of protein-containing organic matter.

### Chemical oxygen demand, dissolved oxygen and phosphate concentration

**Figure 4.**
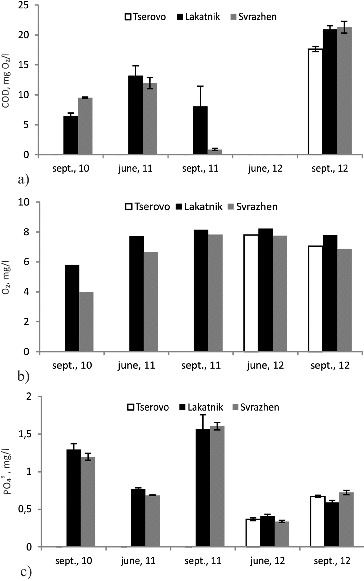
Dynamics of chemical oxygen demand (COD) (a), dissolved oxygen (b) and phosphates (c) in the water of the reservoirs during the studied seasons.

The dynamics of the chemical oxygen demand (COD), dissolved oxygen and phosphate concentrations in the water of the reservoirs during studied seasons are shown in Figure 4. These indicators provide information about the quantity of the carbon compounds; the oxidation potential of the waters (quantity of dissolved oxygen); and the adenosine triphosphate-pool (quantity of the phosphates).

During all the three studied years the values of COD were low, and during the high-flow season in 2012 they were even below the limits of detection. The highest values of COD were measured during the low-flow season in 2012, when the value of COD increased with every sequential reservoir along the course. The high concentrations of ammonium ions and the higher levels of COD during the low-flow season in 2012 show pollution of the waters, probably due to the lack of wastewater treatment plants in the populated places in the region of the cascade. This is confirmed by the slightly lower oxygen concentrations and the higher density of the microbial groups during this season. As a whole during all the three studied years the values of COD fluctuated and did not show mutual dependency among the separate reservoirs or for different seasons and years. Probably because of the small volume of the reservoirs and the constant exchange of water in them the measured value of COD depended entirely on the state of the waters in the Iskar River at that moment.

During the low-flow season of 2010 the lowest oxygen concentrations were registered for the three-year period of study. During the next years the oxygen levels in the reservoirs were higher and with similar values for the different seasons. The measured oxygen showed that the rate of the biodegradation process was quick enough and this rate of self-purification was capable of maintaining high quality of the water.

The dynamics of the phosphate concentration showed that their quantity varied during each season but as a whole their concentration in the separate reservoirs along the water course was constant and did not vary significantly. The highest concentration of phosphate ions was observed again in September 2011, probably due to the intensified processes of biodegradation after the above-mentioned critical situation. The phosphate concentration was higher during the low-flow seasons in all reservoirs. These results are in line with the findings of House and Denison,[15] who observed accumulation of phosphorus during spring and summer low flows and remobilization in autumn. Bowes et al. [16] further highlighted the importance of plant decomposition in the release of phosphorus in the autumn following bio-assimilation in the summer months.

### Microbiological indicators

**Figure 5.**
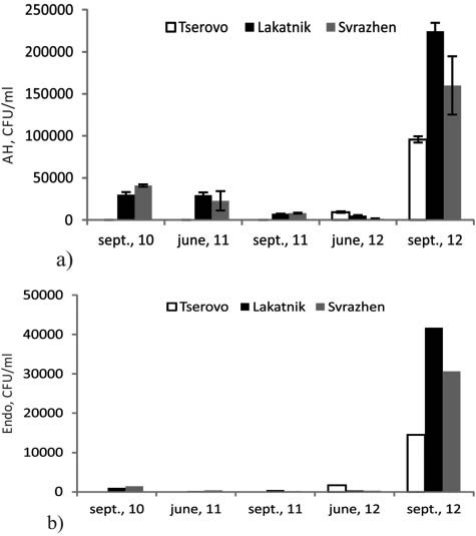
Numbers of aerobic heterotrophic bacteria (a) and bacteria growing on Endo medium (b) in the water of the reservoirs during the studied seasons.

The quantitative parameters of two key microbial indicators were studied in the reservoir water: AH and the complex of Endo bacteria. AH on the one hand show the degree of organic load with biodegradable matter, and on the other hand by their number and dynamics they are an indicator of the speed and scale of self-purification processes. The complex of Endo bacteria is an indicator of faecal contamination and biodegradation potential. These two groups of microorganisms are important for the ecosystem functioning and formation water quality [17,18] and their dynamics in the water of the reservoirs during the studied seasons is given in Figure 5.

In the three reservoirs the density of the microbial heterotrophic complex in the waters was highest during the low water level in 2012, whereas the highest AH counts were scored in the Lakatnik reservoir. These bacterial communities are likely to play a very active role in the rapid *in situ* degradative process.[5,9,19] The great number of microorganisms was in good correlation with the higher values of COD and ammonium ions during the same season. The presence of more pollutants in September 2012 was also proved by the dynamics of the Endo bacteria quantity. Their number was also highest in the Lakatnik reservoir during this season. AH as well as bacteria growing on Endo medium were presented at much lower counts during all other seasons. The higher density of the two types of bacteria during the low-flow season in 2012 can be due to anthropogenic pollution with C- and N-containing organic matter from the populated areas around the cascade or to the construction activities on SHPP Prokopanik in 2012.

In the waters of the three reservoirs from the Middle Iskar cascade there were linear correlations between COD and the two studied groups of microorganisms (COD/АH and COD/Еndo) (Table 2). This is naturally expected, since high concentrations of organic matter normally correspond to an increased microbial number. The linear dependency between COD and AH again confirms that heterotrophic microorganisms are a good indicator of the presence of intensive self-purification processes in the waters of the reservoirs. The correlation between COD and the bacteria growing on Endo medium shows that in most cases the higher values of COD were due to pollutants coming from the populated areas near the Middle Iskar cascade, mainly compounds of protein nature. The lack of any relation whatsoever between the microorganisms and the ammonium ions suggests that the bacteria degrading protein substrates were poorly presented in the studied microbial groups.

**Table 2.** Correlative relations among key hydrochemical and microbiological indicators.

Reservoir	COD/AH	COD/Endo	NH_4_^+^/AH	NH_4_^+^/Endo	PO_4_^3−^/AH
Tserovo			–	–	–
Lakatnik			–	–	–
Svrazhen				–	–

Note: In the table linear correlations among the indicators with a degree of reliability *R* > 60.

## Conclusions

Our three-year study of the self-purification potential of the waters of the reservoirs of three small hydroelectric power plants showed acceleration of the self-purification processes in the region of the Middle Iskar cascade. There was evidence for intensive biodegradation processes most often ending with accumulation of nitrates. Poor management in 2011 caused a critical situation and demonstrated that the lack of adequate measures in the technological management of the SHPP can lead to accumulation of organic matter in the sediments, decreasing the ions of the biogenic elements and reducing the number of microorganisms in the waters. The intensive anaerobic processes in the sediments cause the release of gases (methane, carbon dioxide, hydrogеn sulphide) that are harmful for the environment and slow down the self-purification processes. For the proper ecological functioning of the cascade it is necessary to have adequate management of the sediments accumulating in the reservoirs, depending on the quantities of organic matter in them.
